# The transcription factor ERG increases expression of neurotransmitter receptors on prostate cancer cells

**DOI:** 10.1186/s12885-015-1612-3

**Published:** 2015-08-27

**Authors:** Haydn T. Kissick, Seung T. On, Laura K. Dunn, Martin G. Sanda, John M. Asara, Kathryn L. Pellegrini, Jonathan K. Noel, Mohamed S. Arredouani

**Affiliations:** 1 Department of Surgery, Urology Division, Beth Israel Deaconess Medical Center, Harvard Medical School, 3 Blackfan Circle, E/CLS-446, Boston, MA 02215 USA; 2 Department of Urology, Emory University School of Medicine, Atlanta, GA USA; 3 Department of Medicine, Beth Israel Deaconess Medical Center, Harvard Medical School, Boston, MA USA

## Abstract

**Background:**

The TMPRSS2-ERG gene fusion occurs in about half of prostate cancer (PCa) cases and results in overexpression of the transcription factor ERG. Overexpression of ERG has many effects on cellular function. However, how these changes enhance cell growth and promote tumor development is unclear.

**Methods:**

To investigate the role of ERG, LNCaP and PC3 cells were transfected with ERG and gene expression and metabolic profile were analyzed.

**Results:**

Our data show that expression of ERG induces overexpression of many nicotinicacetylcholine receptors (*nAChRs*). In addition, metabolic profiling by LC-MS/MS revealed elevated production of several neurotransmitters in cells expressing ERG. Consistently, treatment of ERG-expressing cells with nicotine induced elevated calcium influx, GSK3β (Ser9) phosphorylation and cell proliferation. Finally, we show that PCa patientswho are smokers have larger tumors if their tumors are TMPRSS2-ERG gene fusion positive.

**Conclusion:**

Collectively, our data suggest that ERG sensitizes prostate tumor cells to neurotransmitter receptor agonists like nicotine.

**Electronic supplementary material:**

The online version of this article (doi:10.1186/s12885-015-1612-3) contains supplementary material, which is available to authorized users.

## Background

Transcription factor expression and function is commonly disrupted in cancer, leading to the gain or loss of critical characteristics required for tumor development. One such example, found in 50 % of prostate cancers, involves the fusion between the ETS transcription factor ERG with the androgen-regulated gene TMPRSS2, resulting in overexpression of ERG in the prostate epithelium [1]. Overexpression of ERG drives a number of oncogenic effects in prostate cells. For example, ERG co-operates with the PI3K pathway to promote oncogenesis [2, 3]. ERG also increases androgen receptor (AR) binding in mouse prostate, and increases AR transcriptional output in PTEN-deficient prostate cancer patients [4]. ERG-dependent chromatin modification has also been found by many Chip-Seq studies that also revealed that AR binding is significantly altered in ERG-expressing cells [4–6]. ERG may regulate these changes by altering expression of the histone deacetylase, HDAC1, as this is one of the most up-regulated genes in ERG-positive prostate cancer. ERG also induces expression of EZH2, a H3K27 methyl-transferase critically involved in chromatin modification [7]. Additionally, ERG has been found to enhance signaling through the WNT pathway, an event that results in increased activation of β-catenin [8–10]. While these roles suggest an important role for ERG in tumorigenesis, *in vivo* mouse models have found that ERG overexpression alone is insufficient to induce cancer [3, 4]. However, patients with ERG-positive high-grade prostatic intraepithelial neoplasia (HGPIN) are much more likely to progress to prostate cancer [11]. Therefore, the question still remains, if the TMPRSS2:ERG fusion alone is not sufficient to cause cancer, what changes does it induce in prostate epithelial cells to predispose them to become cancerous.

Here we investigated the role of ERG in prostate cancer and found that overexpression of ERG resulted in expression of a number of neurotransmitter receptors. We found that these receptors were functional and treatment of ERG-positive cells with the acetylcholine receptor agonist, nicotine, increased cell growth, calcium signaling and GSK-3β (Ser9) phosphorylation. As a result of this, we measured the polar metabolic profile of ERG-positive cells and found several elevated neurotransmitters. Finally, we found that if patients with TMPRSS2:ERG fusion positive prostate cancer were smokers, they had significantly higher number of biopsy cores positive for cancer, and each core contained a larger proportion of tumor tissue, compared to TMPRSS2:ERG negative smokers, suggesting an increased tumor volume in these patients. Together, these data suggest a novel mechanism by which ERG may contribute to prostate tumorigenesis.

## Materials and Methods

### Patient data & Ethics Statement

Data analyzed in this work was extracted from the BIDMC Early Detection Research Network (EDRN), which enrolled men at 6 clinical practice sites at Harvard, Michigan, and Cornell universities who had undergone their first prostate biopsy were identified. Demographic and pre-biopsy clinical data, including PSA history, digital rectal exam (DRE) results, smoking history, prostate volume and all biopsy procedure details were ascertained on case report forms. Prostate biopsy results were reviewed with reports confirmed by institutional pathologists. Pathology reports included confirmed histology, number of cores and percent of each core involved with carcinoma, and primary and secondary Gleason patterns [12]. Among patients with newly diagnosed (untreated) prostate cancer, TMPRSS2:ERG status was determined using a PCR-based urine TMPRSS2:ERG fusion assay (performed by Gen-Probe, CA) as previously described [13].

All participants had provided written informed consent and all prostate biopsies had been offered according to National Comprehensive Cancer Network (NCCN) guidelines [14].

All research carried out on humans was in compliance with the Helsinki Declaration and approved by the Dana-Farber/Harvard cancer Center Internal Review Board protocol #DF/HCC 05-209.

### Transfection of cells

ERG-RFP or RFP expression was induced in the PC3 and LNCaP cells using a lentiviral transduction system provided by Dr. Owen Witte (UCLA, Los Angeles, CA) as described previously [15].

### Microarray and PCR gene expression analysis

Gene expression was assessed using Affymetrix’s (Santa Clara, CA) GeneChip Human Genome U133 2.0 arrays. Arrays were scanned with the GeneChip Scanner 3000 and GeneChip Operating Software acquired raw fluorescence signal. Scanned array images were analyzed by dChip [16] and CEL file data were normalized using RMAexpress (http://rmaexpress.bmbolstad.com) [17] and annotated using Multiexperiment Viewer software (http://www.tm4.org/mev.html). The data generated have been deposited in NCBI's Gene Expression Omnibus and are accessible through GEO Series accession number GSE55944 (http://www.ncbi.nlm.nih.gov/gds/?term=GSE55944). Primers for select gene subsets were designed using the Primer3 platform (http://bioinfo.ut.ee/primer3/) and analyzed using a StepOnePlus real-time PCR machine from Applied Biosystems (Life Technologies, Grand Island, NY).

### Pathway and Upstream Regulator Analysis

Pathway analysis of the differentially expressed RT-PCR-validated genes (Fig. 2) in ERG- and ERG+ LNCap cells was performed using the commercial systems biology oriented package, Ingenuity Pathways Analysis (www.ingenuity.com). The Score (-log *p*-value) is calculated using Fisher's Exact Test and indicates the likelihood a gene will be found in a network due to random chance. Ingenuity's Upstream Regulator Analysis tool was used to identify the cascade of upstream transcriptional regulators that can explain the observed gene expression changes.

### Metabolomics

Metabolite content measurement in LNCap-ERG^+^ and LNCap-ERG^-^ cells and data analysis was performed as described previously [18–20]. Briefly, 10^6^ cells exponentially growing in media were harvested in 3 mL of cold 80 % v/v HPLC grade methanol. Fresh medium was added 24 and 2 h prior to the extraction. Samples were re-suspended in 20 μL HPLC-grade water for mass spectrometry analysis. 10 μL were injected and analyzed using a 5500 QTRAP triple quadrupole mass spectrometer (AB/SCIEX) coupled to a Prominence UFLC HPLC system (Shimadzu) via selected reaction monitoring (SRM) of a total of 256unique endogenous polar metabolites for steady-state analyses. Peak areas from the total ion current for each metabolite SRM transition were integrated using MultiQuant v2.0 software (AB/SCIEX). Measurements were performed in triplicate and only metabolites that were determined in all 6 samples were retained, normalized and analyzed using MetaboAnalyst2.0 software (http://www.metaboanalyst.ca/MetaboAnalyst/).

### Ca2+ flux

To investigate calcium signaling in prostate cancer cell lines, cells were stained with 1 μM of Fluo-4 AM for 15 min at 37 °C in Hanks Buffered Salt Solution. Cells were then treated with 10 μM solution of nicotine and intracellular Ca^2+^ concentration over time analyzed by flow cytometry (BeckmanCoulter, Galios).

### Pathscan and Western blot analysis of phospho-proteins

Nicotine- or buffer-treated cells were lysed on ice at the various time points with Cell Signaling Technologies cell lysis buffer. Protein concentration was determined using the Thermo Scientific BCA protein assay kit. Analysis of phospho-proteins was performed using Cell Signaling PathScan Akt signaling array (Cat: 9700) as described by the manufacturer’s instructions. Confirmation of PathScan results was performed by western blot. Briefly, protein was separated usingLife Technologies NovexSDS-Page system. Antibodies from Cell SignallingT echnolgy against phospho-GSK3a/b (Ser21/9) (clone 37 F11), β-actin (clone 13E5) and phospho-AKT (Ser129) (clone D4P7F) were used to detect relevant proteins under conditions suggested by the manufacturer.

### Statistics

Statistical analysis was performed using the Student’s *T*-test. *P* values of less than 0.05 were considered significant.

## Results

### ERG overexpression alone induces expression of neurotransmitter receptor genes in LNCaP cells

**Fig. 1:**
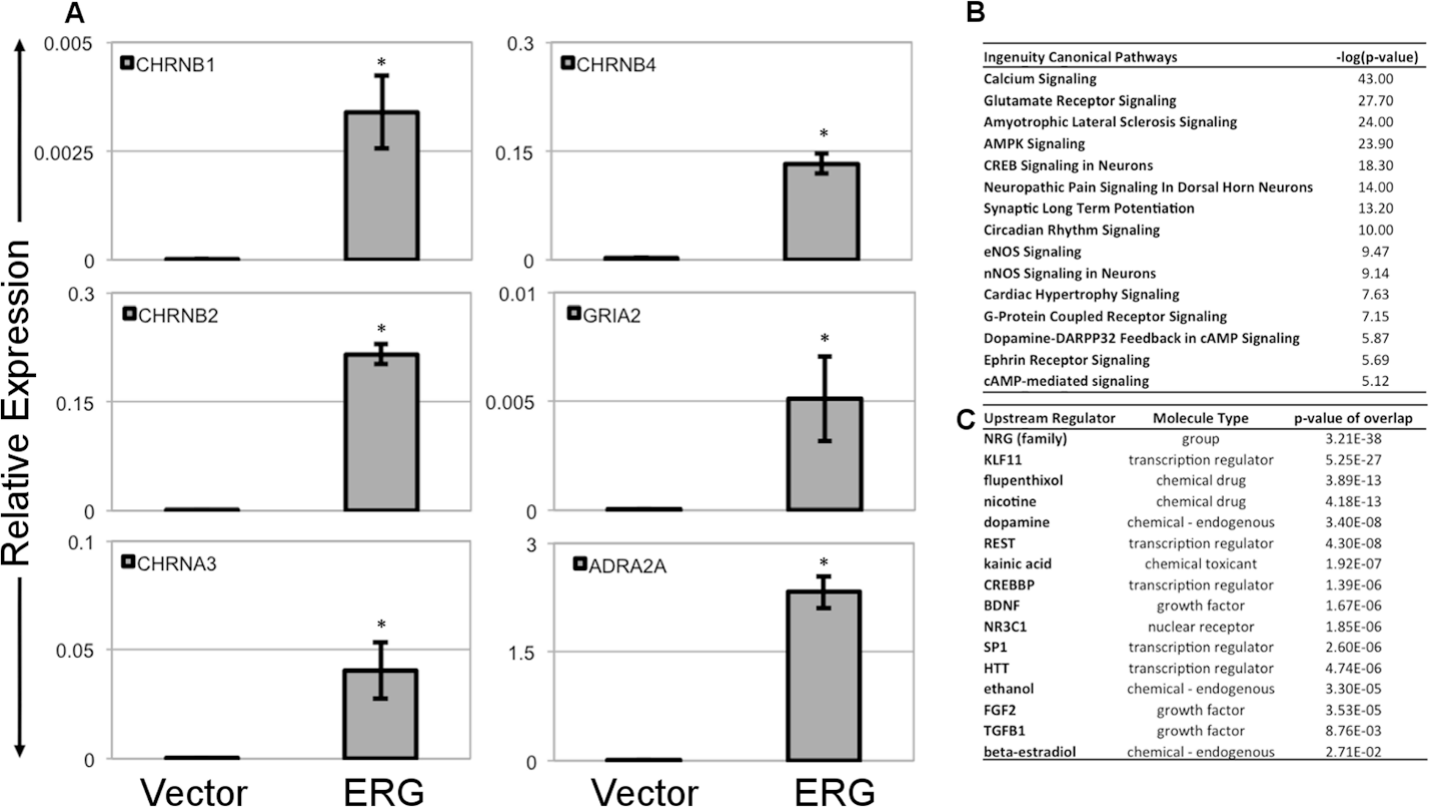
Gene expression of select neurotransmitter receptors in ERG-expressing and control prostate cancer cell lines. **a** PCR analysis of LNCaP cells expressing ERG or vector control. Data is representative of 2 experiments. *P < .05 for ERG vs. Vector control. Sets of differentially expressed neurotransmitter receptor genes (derived from Additional file 1: Table S1) between ERG+ and control LNCap cells as assessed by qRT-PCR were analyzed through the IPA package. Top dysregulated pathways in ERG+ cells (**b**) and predicted alterations in upstream regulators (**c**) are shown

**Fig. 2:**
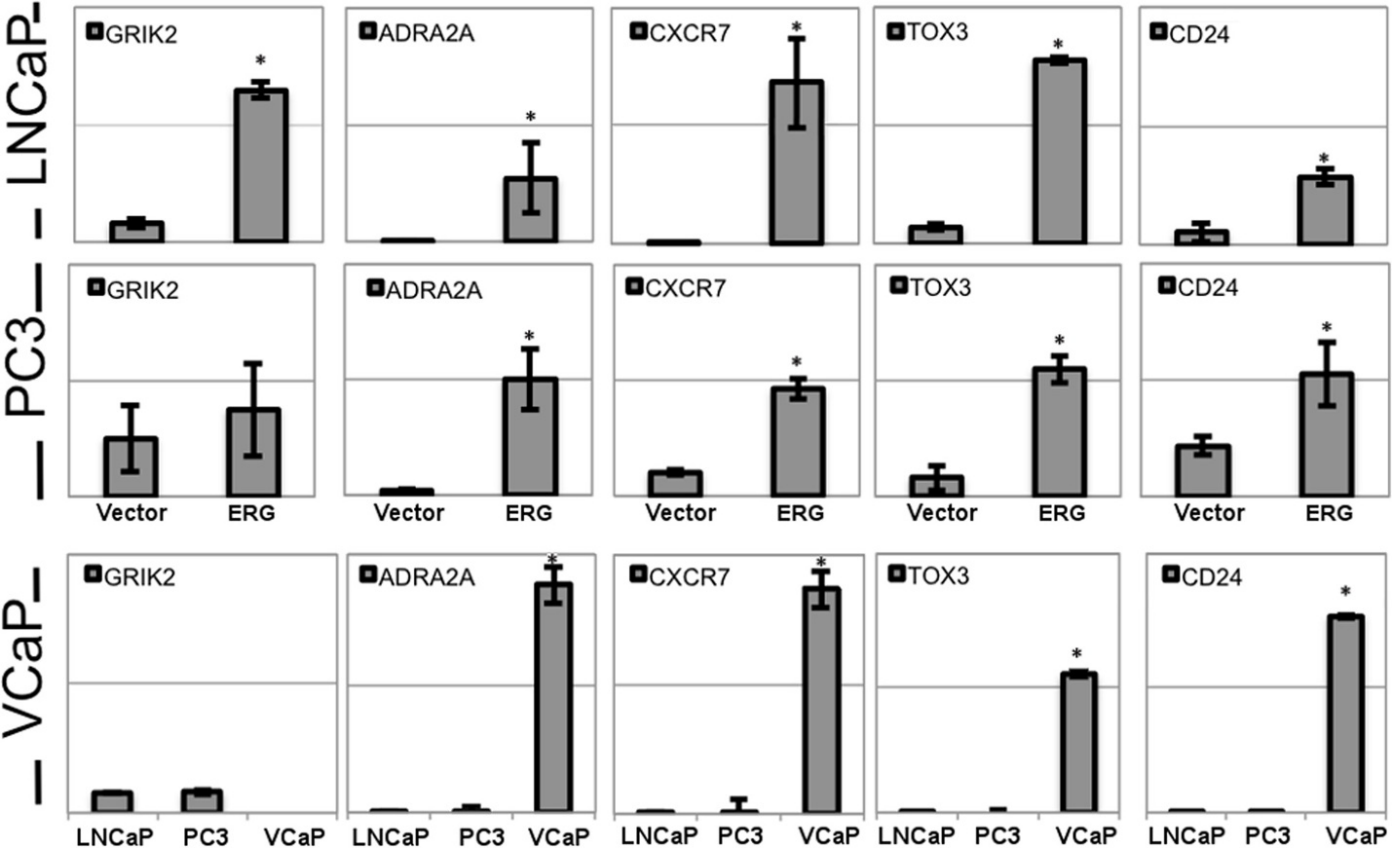
Gene expression in ERG expressing prostate cancer cell lines.qRT-PCR analysis of LNCaP or PC3 cells expressing ERG or vector control. VCaP expression of the same genes analysed compared to WT LNCaP or PC3 cells. Data is representative of 2 experiments. *P < .05 for ERG vs. Vector and Vcap vs. LNCap and PC3

To investigate how expression of ERG alters the function of prostate cancer cells, LNCaP cells were transduced with ERG or a control lentivirus, and transcriptional and metabolic profiling was undertaken. Transcriptional profiling found that amongst the genes up-regulated by ERG were a number of neurotransmitter receptors. To further investigate how expression of neurotransmitter receptors was changed by ERG, we profiled all known acetylcholine, and glutamate receptors by qRT-PCR (See Additional file 1). The nicotinic acetylcholine receptors, CHRNB1, CHRNB2, CHRNB4 and CHRNA3 all increased from an undetectable level in control LNCaP to high levels of expression in LNCaP-ERG cells (Fig. 1a). Similarly, the adrenergic receptors, ADRA2A and ADRA1D, and the glutamate receptors, GRIA2 and GRIN1 were all highly up-regulated in the ERG-positive cells (See Additional file 1: Table S1 for full neurotransmitter expression data). Similar increase in expression of these genes was observed in ERG-positive PC3 cells and additionally, VCaP cells express far greater levels of all these neurotransmitter receptors than either wild-type LNCaP or PC3, further supporting the hypothesis that ERG induces expression of these genes. To predict the potential implications of CHRN receptor overexpression in LNCap cells, we used the qRT-PCR data mentioned above to perform an *in silico* analysis using the Ingenuity Pathway Analysis tool. Our analysis revealed significant dysregulations in many known neuronal signaling pathways. These include Calcium Signaling, Glutamate Receptor signaling, Neuropathic Pain Signaling In Dorsal Horn Neurons, Synaptic Long Term Potentiation, G-Protein Coupled Receptor Signaling, and Dopamine-DARPP32 Feedback in cAMP Signaling (Fig. 1b). Likewise, upstream regulator analysis of the same PCR data identified patterns that mimic the action of nicotine, dopamine, kainic acid, flupenthixol, ethanol, beta-estradiol and Brain-derived neurotrophic factor (Fig. 1c). In addition to these neurotransmitter receptors, several other genes associated with nervous system were upregulated including CD24, the neural stem cell marker was one of the most highly up regulated genes, as was the neural transcription factor TOX3 (Fig. 2). These genes were also increased in PC3 cells transfected with ERG, as well as being highly expressed in VCaP, a cell line that naturally expresses the TMPRSS2:ERG fusion (Fig. 2).

### ERG expression significantly alters the metabolic profile in LNCap cells

**Fig. 3:**
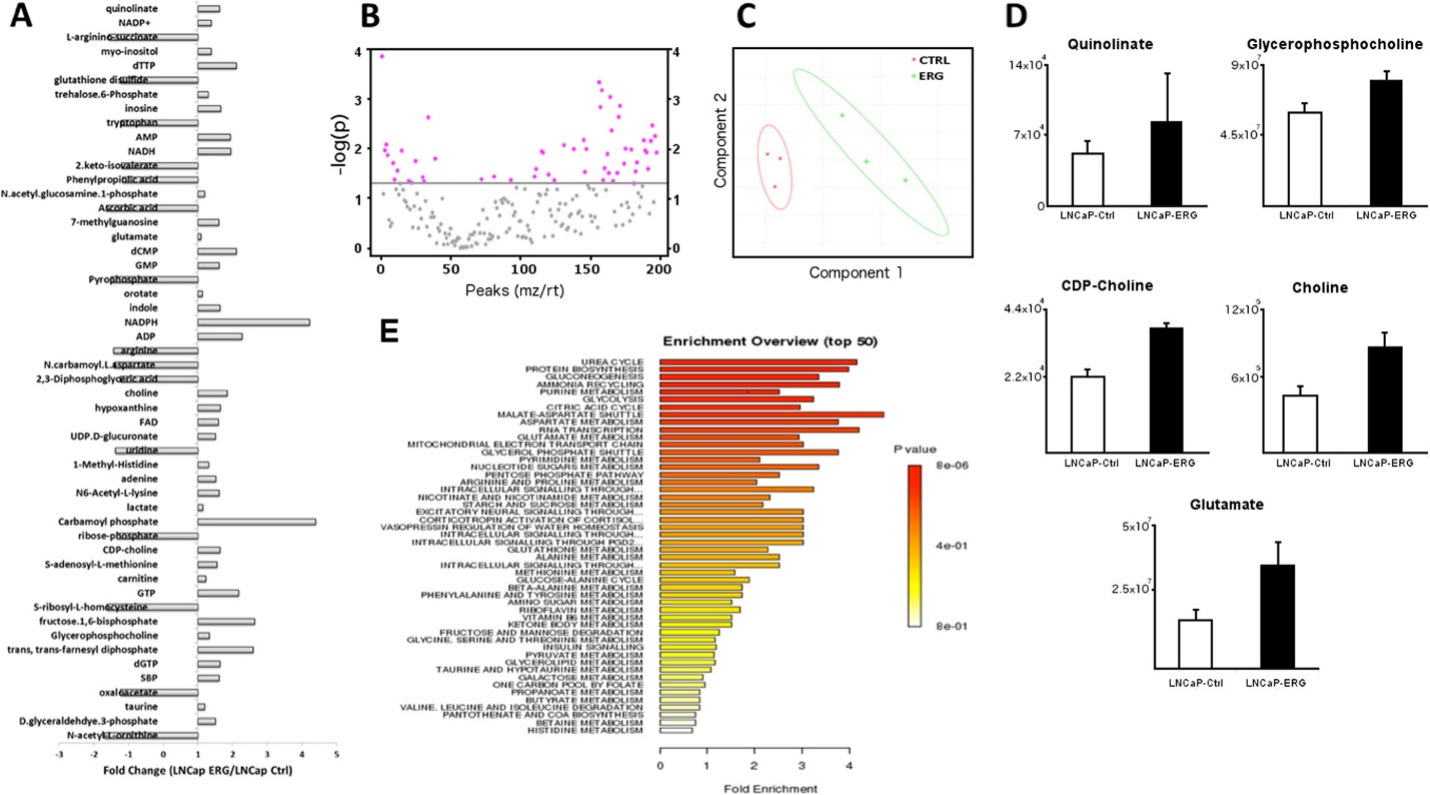
Metabolite and Neurotransmitter production is elevated in LnCap and PC3 cells transfected with ERG. Among the metabolites (see Methods Section) measured by LC-MS/MS, 52 were found to be significantly regulated in ERG+ LNCap as compared to ERG- LNCap (*P* < 0.05). Fold change in production is shown in (**a**) and the *t* test in (**b**). Principal component analysis (PCA) provided evidence for clearly distinct metabolic phenotypes comparing ERG+ LNCap and ERG- LNCap cells (**c**). The 52 metabolites include the neurotransmitters quinolinate, glycerophosphocholine, CDP-choline, choline, and glutamate (**d**). Enrichment analysis identified many metabolic pathways in which the 52 differentially produced metabolites play a role (**e**)

To further investigate changes in the prostate cancer cells after ERG induction, we analyzed the metabolic profile of the cells using high-throughput tandem mass-spectrometry (LC-MS/MS) [18]. 256 metabolites were targeted using this method and 52 of these were significantly regulated in the ERG expressing cells (Fig. 2c). Additionally, ERG-positive LNCaP cells clustered significantly compared to controls (Fig. 2b). Energy production and cellular proliferation pathways were enriched in the ERG-positive cells including protein biosynthesis, gluconeogenesis and purine and pyrimidine metabolism (Fig. 3d). Most importantly, metabolites belonging to the excitatory neural signaling pathway were increased. In particular, ERG-positive LNCaP cells had a significant increase in the neuro-transmitting molecules, quinolinate, choline and glutamate (Fig. 3e). Additionally, two other members of the acetylcholine synthesis pathway, CDP-choline and glycerophosphocholine, were significantly increased. Together, these data suggest that ERG induces an increase in expression of neuro-transmitter receptors and production of neuro-transmitting molecules.

### Nicotine enhances growth of ERG-positive prostate cancer cells and induces Ca^2+^ influx and phosphorylation of GSK3β

**Fig. 4:**
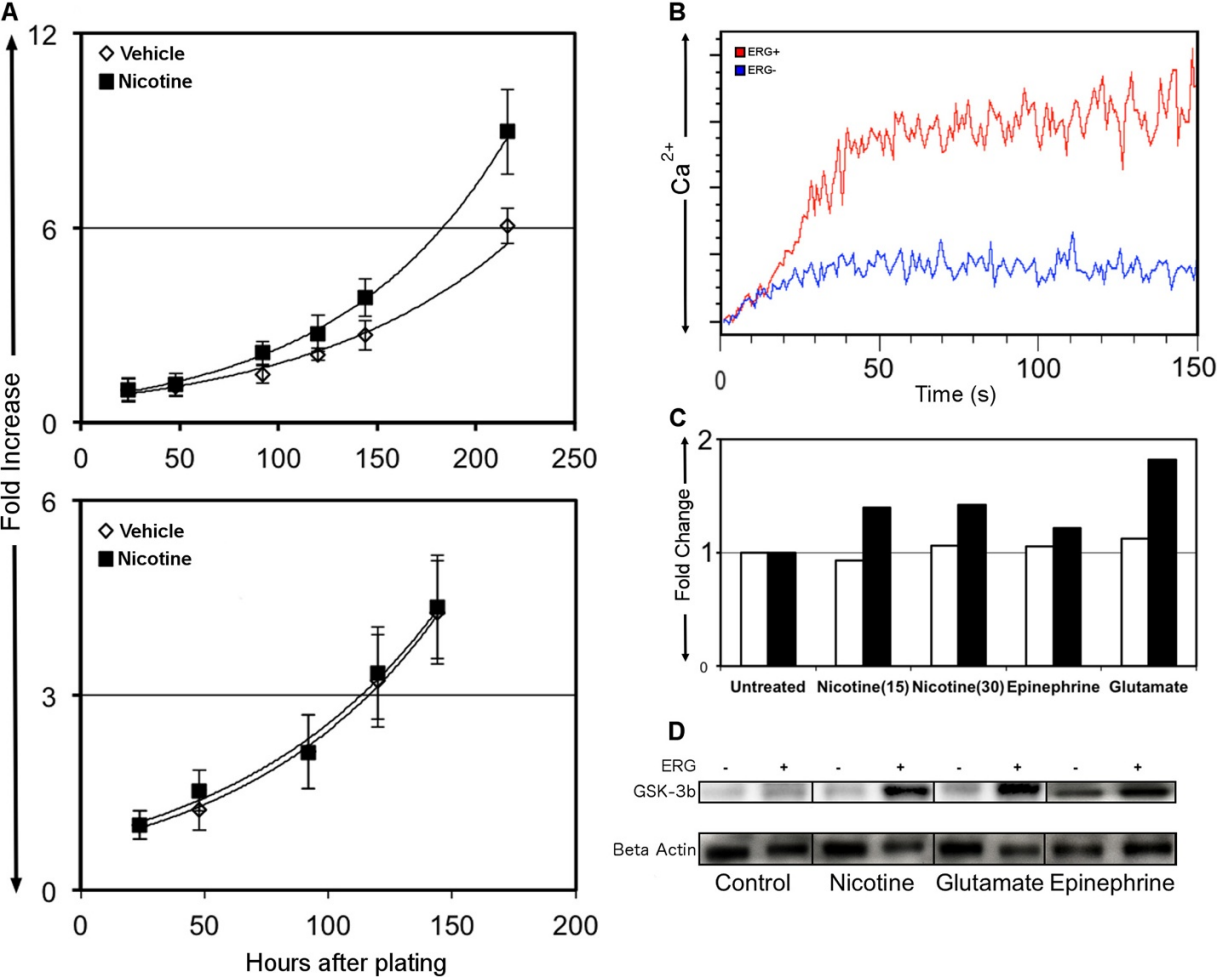
Treatment of LnCaP-ERG cells with nicotine enhances cell growth rate, calcium flux and phosphorylation of GSK-3β. **a** LNCaP cells transfected with ERG or a control Vector were treated with nicotine and growth analysed by cell imaging cytometry. **b** Calcium Flux in cells treated with nicotine. LNCaP cells were stained with Fluo-4 AM. Cells were then treated with nicotine and Calcium determined by flow cytometry. **c** Phospho-protein analysis of nicotine treated cells. Protein was extracted from LNCaP-ERG or LNCaP vector cells treated with nicotine, epinephrine or glutamate and analyzed by Pathscan (**c**) and Western Blot (**d**)

**Fig. 5:**
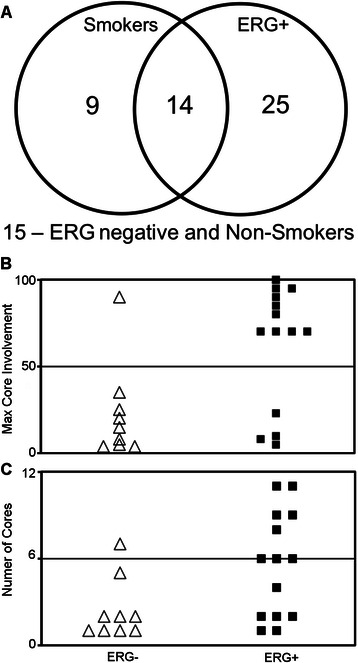
Prostate cancer patients positive for ERG have larger tumors if they are also smokers. **a** Venn Diagram showing smoking history and urine PCR-determined TMPRSS2:ERG gene fusion status of prostate cancer patients from the prostate cancer EDRN cohort. **b**, **c** Histopathological analysis of TMPRSS2:ERG+ tumors from smokers showed a higher number of biopsy cores involved and a higher proportion of core involvement, compared to TMPRSS2:ERG- tumors

Given the over-expression of numerous acetylcholine receptors, we hypothesized that prostate cancer cells would be more responsive to acetylcholine receptor agonists. We investigated the role of nicotine in enhancing the growth of ERG-positive LNCaP cells. ERG-positive LNCaP cells were treated with 10 nM of nicotine or vehicle control, and the number of cells was counted at 24-h intervals using cell imaging cytometry. Growth of the LNCaP-ERG cells treated with nicotine was significantly faster than controls with an average doubling time of 59 h compared to 72 h for controls. In contrast, ERG negative cells had no significant change in growth when exposed to nicotine (Fig. 4a). Given these change in growth kinetics of ERG expressing cells in response to nicotine, we investigated downstream signaling events of nicotine exposure. Nicotine treatment induced a significant increase in intracellular Ca^2+^ after 30 s in ERG expressing cells compared to normal LNCaPs (Fig. 4b). To further investigate signaling events, protein isolated from ERG-positive cells treated with nicotine, glutamate or epinephrine was analyzed with a multiplex phospho-signaling array. Of the 18 different proteins measured, only phospho-GSK3βwas increased at all time points following nicotine exposure (Fig. 4c, Additional file 1: Data 2 for all phosphorylation data). Additionally, other neurotransmitter receptor agonists Epinephrine and Glutamate increased GSK3β phosphorylation. Phosphorylation of GSK3βwas further investigated by western blot and a similar response to all neurotransmitters found (Fig. 5d). These findings indicate that over-expression of ERG confers the ability on prostate cancer cells to respond to acetylcholine receptor agonist through normal GPCR signaling pathways.

### TMPRSS2:ERG^+^ prostate cancer patients that smoke have a larger proportion of biopsy core involvement

Given our findings indicating that ERG-positive prostate tumor cells exhibited expression of neurotransmitter receptors and that nicotine enhanced tumor cell growth *in vitro*, we hypothesized that growth of ERG-positive prostate cancer would be enhanced in patients that smoked. We used data collected from a cohort of patients enrolled through the Early Detection Research Network (EDRN) to correlate biopsy core involvement to ERG and smoking status. Among patients with newly diagnosed (untreated) prostate cancer in this cohort, 62 of these patients had their TMPRSS2:ERG status determined using a urine TMPRSS2:ERG fusion assay (See Additional file 1: Data #1 for all patient data). Of these 62 patients tested, 24 reported being regular smokers and 39 were positive for ERG (Fig. 5a). Interestingly, smokers whose tumors had the TMPRSS2:ERG fusion had a significantly higher number of both biopsy core involvement and total core involvement, compared to patients whose tumors are fusion negative (Fig. 5b and c). Similarly, given a patient was positive for ERG, if they also smoked they had a near significant (0 = 0.055) but no difference in number of cores involved. In contrast, non-smokers had no significant difference in either of these measurements between ERG^-^ and ERG^+^ cancers. No significant difference in Gleason score or T staging was observed between the ERG groups. These data show *in vivo* evidence supporting genomic data that suggested that ERG induced expression of neurotransmitter receptors in prostate cancer cells, and a specific mechanism by which ERG may contribute to tumor development.

## Discussion

Expression of ERG is an early event in prostate cancer tumorigenesis, but alone is insufficient to induce cancer (3, 4). Here we investigated how ERG changed the function of prostate cancer cells by transducing LNCaP and PC3 cells with ERG, and measuring changes in gene expression and metabolic profiles. Amongst the set of genes most significantly changed, we found that expression of ERG in LNCaP and PC3 cells caused up-regulation of acetylcholine and adrenergic receptors (Fig. 1). Additionally, metabolic profiling of these cell lines revealed an increase in the concentration of excitatory neural signaling molecules, choline, glutamate and quinolinate (Fig. 3). To test the functionality of the neuro-transmitter receptors, we treated cells with the acetylcholine receptor agonist, nicotine. Signaling through these receptors induced an increase in Ca^2+^ release, phosphorylation of GSK3β (Ser9) and cell proliferation (Fig. 4). GSK3β phosphorylation at Ser9 is a downstream event of many signal pathways including WNT, AKT, and Ca^2+^ signaling [21]. GSK3β has many effects that could contribute to tumor development. This includes its central function as a regulator of glucose metabolism and cell proliferation [21] and we found that metabolites within both gluconeogenesis and the citric acid cycle were two of the most enriched pathways of ERG expressing cells (Fig. 4). Additionally, metabolites involved in protein biosynthesis, purine metabolism, and pyrimidine metabolism were all increased in ERG expressing cells, suggesting a higher metabolic rate in these cells (Fig. 3). ERG has previously been reported to control phosphorylation of GSK3β (Ser9) by regulating the WNT pathway [8]. However, our data suggests a possible novel mechanism by which ERG regulates GSK-3β; up-regulation of neuro-transmitter receptors leading to an inappropriate response to local neurotransmitters. In addition to these general roles, GSK3βhas been suggested as a contributing factor to prostate cancer cells gaining androgen independence [22]. Together, these findings suggest that ERG may contribute to tumorigenesis through upregulating neurotransmitter receptors and increasing GSK3β phosphorylation and further downstream signaling events.

Given our findings of the increased growth response to nicotine *in vitro*, we investigated whether ERG had a similar effect on increasing susceptibility to nicotine in PCa patients. We found that patients who smoked and had TMRPSS2:ERG positive prostate cancer had a significantly higher number of biopsy cores positive for cancer, and these cores had a higher proportion of cancer involvement (Fig. 5). We acknowledge that the size of this dataset is very small, and the lack of data regarding the quantity and duration of the patients smoking habits, or other unknown confounding variables, make any certain claims about smoking leading to worse outcomes for prostate cancer patients who are ERG positive premature. However, this effect has been reported in many other cancers, in particular in lung cancer where nicotinic receptors regulate proliferation and inhibit apoptosis [23, 24]. More recently, similar effects have been described in bladder, breast and colon cancer [25, 26]. Based on the findings of other cancers and our *in vitro* data, we believe that this patient data carries more weight than an epidemiological study of this size usually would, and suggests that larger epidemiological studies should investigate the link between ERG-positive prostate cancer, smoking and prostate cancer disease outcome.

## Conclusions

In summary, our data indicates that enhanced neurotransmitter receptor expression in ERG-expressing cells translates into elevated responsiveness to exogenously derived molecules like nicotine, or endogenous molecules, glutamate and epinephrine. Also, in silico analysis of the receptor expression data suggests that a similar responsiveness might also develop towards other endogenous molecules known to act on neurons, including dopamine, kainic acid, flupenthixol, ethanol, β-estradiol and Brain-derived neurotrophic factor. It is therefore possible that these molecules might negatively affect ERG^+^PCa if they are present in the tumor microenvironment. Together these data indicate that ERG has effects on cell metabolism and proliferation by increasing cell responsiveness to exogenous neurotransmitter receptor agonists such as nicotine, and possibly to endogenous neurotransmitters.

## Additional file

Additional file 1: **ERG-dependent neurotransmitter receptor expression, pathscan data, and patient tumor pathology scoring.** (XLSX 100 kb)

## Abbreviations

AR: Androgen receptor
DRE: Digital rectal exam
EDRN: Early Detection Research Network
HGPIN: High-grade prostatic intraepithelial neoplasia
PCa: Prostate cancer
nAChRs: Nicotinicacetylcholine receptors
NCCN: National Comprehensive Cancer Network
